# Determinants of stakeholders’ attitudes and intentions toward supporting the use of *Wolbachia*-infected *Aedes* mosquitoes for dengue control

**DOI:** 10.1186/s12889-021-12166-w

**Published:** 2021-12-23

**Authors:** Ahmad Firdhaus Arham, Latifah Amin, Muhammad Adzran Che Mustapa, Zurina Mahadi, Mashitoh Yaacob, Maznah Ibrahim

**Affiliations:** 1 Pusat Pengajian Citra Universiti, Universiti Kebangsaan Malaysia (UKM), UKM, 43600 Bangi, Selangor Malaysia; 2 The Institute of Islam Hadhari (HADHARI), Universiti Kebangsaan Malaysia, UKM, 43600 Bangi, Selangor Malaysia

**Keywords:** Attitudes, Intentions, Predictors, PLS-SEM, *Wolbachia*-infected *Aedes* mosquito, Malaysia

## Abstract

**Background:**

A recent approach in controlling dengue is by using the *Wolbachia*-infected Aedes mosquito (WiAM). The approach has been reported to be more effective than traditional methods, such as fogging. Therefore, it is imperative to assess the factors predicting its acceptance among stakeholders before implementing this technology more widely in Malaysia.

**Methods:**

The survey data were collected from two primary stakeholder groups using a stratified random sampling technique. The two primary stakeholder groups were scientists (*n* = 202) and the public (*n* = 197) in the Klang Valley region of Malaysia, a hot spot area known for the high rate of dengue cases. The respondents answered questions on a seven-point Likert scale survey regarding trust in key players, attitudes toward nature versus materialism, religiosity, perceived benefits, perceived risks, attitudes, and intentions. The data were analyzed using Smart Partial Least Square (SmartPLS) software (version 3.2.6) to determine the predictors influencing attitudes and intentions to support the use of WiAM technology.

**Results:**

The results indicated a strong positive relationship between attitudes and intentions to support the use of WiAM (*β* = 0.676, *p* < 0.001). The most important significant predictor for attitudes was perceived benefits (*β* = 0.493, *p* < 0.001), followed by perceived risks (*β* = − 0.080, *p* = 0.048). Trust in key players, attitudes toward nature versus material, and religiosity had indirect relationships with attitudes through the perceived benefits and risks.

**Conclusions:**

The identified predictors can serve as indicators for the decision-making process regarding WiAM implementation in Malaysia and other developing countries with similar demographics and cultures.

**Supplementary Information:**

The online version contains supplementary material available at 10.1186/s12889-021-12166-w.

## Background

*Wolbachia*-infected *Aedes* mosquito (WiAM) is a biological method that has been introduced as an alternative technology to control dengue disease [55]. The technology works by infecting the male *Aedes* mosquitoes with the bacteria known as *Wolbachia* [31]. *Wolbachia* is one of the most effective insect symbionts, primarily due to its ability to manipulate insect reproduction and its association with major human pathogens, providing potential opportunities for disease control [20, 86]. The male *Aedes* mosquitoes are initially infected with *Wolbachia* through a laboratory procedure that induces cytoplasmic incompatibility, limiting viable eggs production in uninfected female mosquitoes [60]. The male WiAMs are then released into the environment to mate with uninfected female Aedes mosquitoes. Due to cytoplasmic incompatibility, the fertilized eggs will not hatch because the embryos formed in the female mosquitoes die. Thus, the wild mosquitoes’ fertility rate and population size decrease [50]. Rapid invasion of the insect’s host population is likely to take place as *Wolbachia* are maternally inherited. Thus, WiAM potentially inhibits the transmission of the dengue virus.

In a laboratory experiment, *Wolbachia* was found to have successfully block dengue transmission in at least 37.5% of Aedes mosquitoes after 14 days of infection [18]. Laboratory results also showed *Wolbachia*’s positive effect in inhibiting dengue virus transmission when an open release of *Wolbachia*-infected mosquitoes was introduced into the wild Australian Aedes population with the Australian Pesticides and Veterinary Medicines Authority’s approval [APVMA permit 12,311] [51]. Staggered open release of WiAM has inhibited dengue transmission in Cairns and surrounding locations in northern Queensland, Australia, between 2016 and 2017 [79]. As WiAM has been demonstrably effective in inhibiting dengue virus transmission in other countries [33, 49, 50, 65, 83, 86, 89], it has immense potential to suppress dengue transmission in Malaysia. For instance, Nazni et al. [70] reported reduced dengue cases in Kuala Lumpur, where *Aedes aegypti* mosquitoes carrying wAlbB *Wolbachia* were released. In collaboration with the Malaysian Ministry of Health, further field trials are continuing in diverse areas.

Several field tests discovered a clear association between WIAM technology and decreasing rate of dengue cases locally and globally. However, the technology has not been widely tested or used. Similarly, the details concerning stakeholders’ acceptance of this technique remain unknown. Previous studies have shown that consumers’ acceptance of new technology tends to be conditional and depends on several factors. Nevertheless, Arham et al. [12, 13] reported that stakeholders in Malaysia cautiously expressed a positive attitude toward WiAM technology, and several relevant factors influenced their attitudes. Therefore, before implementing WiAM technology extensively in Malaysia, understanding predictive factors that influence stakeholder’s acceptance of WiAM technology is crucial. The present study aimed to elucidate the relationship between the predictive factors of stakeholder’s attitudes and intention to support WiAM technology usage and the parties implementing the technology.

## Theoretical framework & development of hypotheses

This study is a continuation of Arham et al.’s work which utilized mean scores and regression analyses to determine factors predicting attitudes toward using WiAM technology [12, 13]. However, linear regression has the limitation of analyzing only one layer of relationships between the independent and dependent variables at a time. Further studies were suggested to undertake structural equation modeling (SEM) to simultaneously analyze and determine the relationships between the predicting factors [14]. The present study framework was developed from models formulated by Bredahl [21], Pardo et al. [72], Bronfman et al. [22], and Amin and Hashim [8], which were based on [30]. According to Fishbein [30], a person’s attitude toward an object is the sum of beliefs they have about the attributes or consequences of the object, generally cited as outcome beliefs and evaluations.

**Fig. 1:**
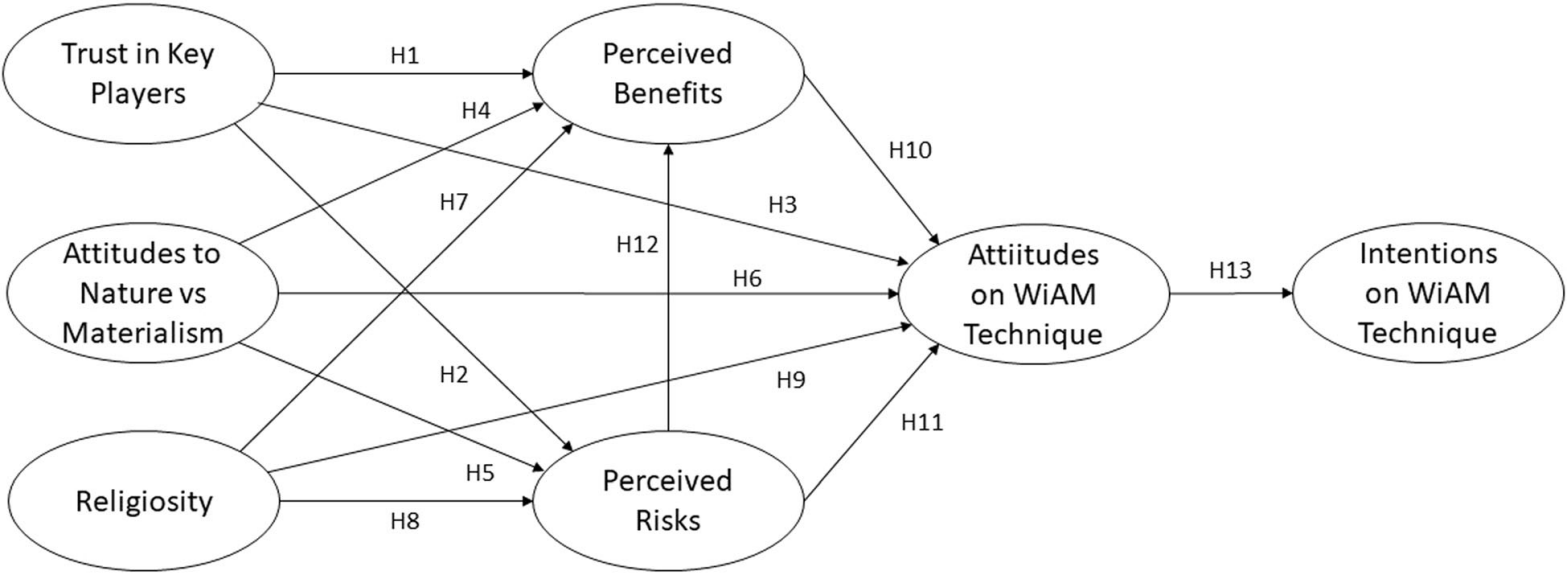
Research Framework for Stakeholders’ Attitudes and Intentions toward Supporting the Use of WiAM Technology

The current study framework grouped variables based on their potential influence (Refer to Fig. 1). In addition, the proposed model comprises four constructs: general factors, specific factors, attitudes, and intentions. General factors include trust in key actors, attitudes toward nature versus materialism, and religiosity, while specific factors encompass perceived benefits and risks. Both exogenous constructs are associated with the endogenous constructs represented by attitudes. Attitudes subsequently act as an exogenous construct linked to endogenous constructs for the intention factor.

### Trust in key Players

Trust is the foundation for maintaining relationships and balancing the roles of stakeholders and implementers or researchers [90]. The trust concept encompasses how a person believes in something without any conditions [76]. Trust results in the stakeholders’ willingness to have a particular party as the primary source for distributing necessary information [68]. In other words, trust is an essential factor influencing stakeholders’ attitudes toward accepting something as beneficial, especially in evaluating new technologies [16, 19, 35]. Trusting the particular party’s responsibility causes stakeholders to accept any risks or dangers resulting from new technology [22]. This particular aspect of trust has a significant positive relationship with stakeholders’ attitudes toward biodiesel products [7] and stakeholders’ willingness to accept biobanking technologies [11]. Due to the importance of this factor in assessing attitudes and intentions toward supporting the WiAM technology usage, the following hypotheses were proposed:

*H1: When stakeholders trust key players, they will perceive more benefits associated with WiAM technology.*

*H2: When stakeholders trust key players, they will perceive fewer risks associated with WiAM technology.*

*H3: When stakeholders trust key players, they have a more positive attitude toward WiAM technology.*

### Attitudes toward nature versus materialism

Attitudes toward nature versus materialism are essential factors in whether stakeholders are more inclined to appreciate nature or be materialistic [6]. Regarding the current study, attitudes toward nature versus materialism refer to stakeholders’ attitudes toward new technologies. For instance, they may feel that the technology presents environmental risks or has certain advantages [37, 77]. Amin et al. [9] stated that a person who is more supportive of biotechnology is more inclined toward materialism. Respondents inclined toward nature tend to be more cautious about accepting modern technology [12, 13]. For instance, Amin and Hasrizul (2015) identified significant relationships indicating that more likely-to-be materialistic stakeholders perceive less risk toward genetically modified mosquitoes’ technology [8]. Other studies have also found that stakeholders prioritizing materialistic values tend to perceive the benefits of xenotransplantation development to be less risky [6]. Due to the importance of attitude toward nature versus materialism in assessing attitudes and intentions toward supporting WiAM technology usage, three hypotheses were proposed:

*H4: When stakeholders are more inclined toward material values, they will perceive more benefits of WiAM technology.*

*H5: When stakeholders are more inclined toward material values, they will perceive fewer risks of WiAM technology.*

*H6: When stakeholders are more inclined toward material values, they will have a more positive attitude of WiAM technology.*

### Religiosity

Religiosity refers to beliefs, rituals, and community involvement in god-related religions that symbolize the community’s identity and drive their behavior [1]. Fam et al. [27] described religiosity as an abstract concept where each religion has its own understanding and beliefs. Religiousness can be assessed subjectively through spiritual experience [46] or the orientation of belief and adherence to religion in determining one’s life course (Worthington Jr. et al., [91]). According to Sheth and Mittal [84], religiosity refers to the human social life rules that encompass the aspects of belief, devotion, and human ability to his god. Religiosity is an influential factor that may shape public opinion on their lives [4]. The Malaysian public places an extremely high value on religion, and the tendency to accept any new technology depends on their religiosity level [6, 10]. Based on the importance of religiosity in assessing attitudes and intentions toward supporting the WiAM technology usage, the following hypotheses were proposed:

*H7: When stakeholders are more religious, they will perceive more benefits associated with WiAM technology.*

*H8: When stakeholders are more religious, they will perceive fewer risks associated with WiAM technology.*

*H9: When stakeholders are more religious, they will have a more positive attitude toward WiAM technology.*

### Perceived benefits and risks

Acceptance of new technologies depends on how the perceived benefits and risks determine attitudes and intentions [34, 38, 66–69]. Rowe [78] stated that perceived benefits and risks are essential factors in predicting community acceptance. However, they are complex to be understood individually because these factors have consistently been inversely related [29, 36, 45]. Alhakami and Slovic [3] asserted that the relationship between perceived benefits and risks is reciprocal because the association is assessed in a bipolar fashion, whether good or bad, dangerous or non-hazardous, scary or non-scary. Four hypotheses were proposed due to the importance of perceived benefits and risks as factors influencing attitudes and intentions toward supporting WiAM technology usage:

*H10: When stakeholders perceive more benefits associated with WiAM technology, their attitude toward the technology will be more positive.*

*H11: When stakeholders perceive more risks associated with WiAM technology, their attitude toward the technology will be less positive.*

*H12: When stakeholders perceive more risks associated with WiAM technology, they will perceive fewer benefits associated with the technology.*

### Attitudes and intentions

Attitudes represent the belief individuals refer to as behavioral probabilities of producing the desired outcome, whether or not it is profitable [40, 64]. Conversely, intentions to act are determined by the attitude and subjective norm to determine the actual behavior [40]. The attitude component is viewed as a crucial factor linking the relationship between the intention of distal and proximal factors to support. An attitude is based on relatively social beliefs, feelings, and tendencies toward important subjects, collections, events, or symbols [52]. Besides, attitude also describes whether an individual likes or dislikes an object through an evaluation process to determine whether the individual’s behavioral intentions are positive or negative [62, 63, 92].

Acceptance of new technologies depends mainly on the people’s support, reflecting their intentions to use the technology [66, 67, 73]. Intentions are also a motivating factor in influencing any behavior [17] that predicts positive relationships in shaping actions [81]. In the health care context, attitudes and intentions to implement the WiAM technology are considered one of the most critical dimensions to evaluate social acceptance in combating dengue. Due to the importance of attitudes and intentions toward supporting WiAM technology usage, the following hypothesis was proposed:

*H13: When stakeholders have more positive attitudes toward WiAM technology, they will have higher intentions to support the technology.*

## Methods

Klang Valley region in Malaysian was chosen as the target location for this study to determine stakeholders’ attitudes and intentions toward supporting the use of WiAM technology. This area was mainly selected because the region has the highest number of dengue cases, as reported by the Malaysian Ministry of Health (http://idengue.arsm.gov.my/) [2]. The sample comprised adult respondents above 18 years old, while data collection was undertaken from September 2016 to September 2017. The respondents were selected using a simple stratified random sampling technique and divided into scientists and the public. The researchers and trained enumerators subsequently administered a survey face-to-face. The group of scientists (e.g., academicians, government officers, health officers, scientists, researchers, and postgraduate students in the field of dengue) were selected because they play a crucial role due to their direct involvement in research on dengue or the control of dengue in Malaysia. The public was selected due to their exposure to dengue.

Besides, the introduction of any dengue control technology requires their approval. Combining these two major stakeholder groups for the modeling process is essential in determining the WiAM technology acceptance. The data for both groups were combined due to their similar interests and roles as primary potential beneficiaries of the WiAM technology. The combined model provides an initial picture of the relationships among the factors predicting stakeholders’ attitudes and intentions to adopt WiAM. However, generalizing the model beyond this population is not recommended. Therefore, further studies involving multi-group analyses and cross-validation of the model involving other stakeholder groups and regions should be undertaken.

The sample size in this study was calculated using the ‘10 times rules’ recommended by Hair et al. [44]. The research instrument comprised 41 items. Therefore, 410 respondents were required to fulfill the sample size. Nevertheless, this sample size surpassed the minimum number suggested by the G*Power 3.1.9.2. Faul et al. (2009) recommended using the G*Power 3.1.9.2 software to conduct a statistical power analysis for social and behavioral sciences. By utilizing this software to calculate the sample size at a statistical power of 0.80 [25, 28], a medium effect size (f = 0.15), and a significance level of 0.05, a sample of 277 respondents was proposed. After considering the possibility of incomplete data, 415 questionnaires were distributed. Nevertheless, only 399 questionnaires were retained involving scientists *(n* = 202) and the public (*n* = 197) after the data cleaning process.

A brief, unbiased introduction to WiAM technology was presented to the respondents before they completed the questionnaires. Kelley [56] recommended adopting this approach to assess the attitude of sophisticated and unsophisticated respondents on complex issues. Therefore, the respondents do not need to have any prior knowledge about the new technology. Conversely, the provision of information beforehand does not affect people’s attitudes to new technology [85]. The use of multi-indicators for the constructs also reduces measurement errors (1995).

A multidimensional instrument was developed based on a validated study by Amin and Hashim [8] to measure the factors, attitudes, and intentions predicting support for WiAM. To measure each item, the respondents responded to their agreement on a seven-point Likert scale ranging from 1 = strongly disagree to 7 = strongly agree. The content validity of the instrument was assessed by seven experts in environmental management, sustainability governance, environmental health and science, and consumer behavior involved in dengue control and prevention. The instrument was prepared in Malay and translated into English to enable the respondents to respond in a familiar language. Two language experts validated the two-way translation. The questionnaire was subsequently distributed to 126 respondents for a pilot test by several trained enumerators. Furthermore, the data obtained were then tested for validity and reliability, and the wordings for ambiguous items were refined. The actual field study was subsequently conducted face-to-face with the respondents to explain any misunderstandings directly. The English version questionnaire used in this study was placed in supplementary file.

According to the Guidelines for the Ethical Review of Clinical Research or Research for human subjects by the Medical Review and Ethics Committee (MREC), Ministry of Health Malaysia, the public behavior questionnaire analysis and informed consent were waived from MREC approval. The participants were subjected to minimal risk, and the study contained only publicly accessible data. However, informed consent was obtained verbally without collecting identifiable private information to acknowledge the respondents’ participation. The questionnaire was marked with the respondent’s number, and only general demographic questions required completion.

The data can be obtained from the Mendeley Data repository by Arham and Amin [15] and the corresponding author’s data storage. The data were analyzed using SmartPLS-SEM software (version 3.2.7) to determine the predicting factors and their relationships, as Ringle et al. [75] recommended. The PLS-SEM is recommended for this study context due to its exploratory nature. This method is extremely useful in justifying the use of multiple factors to explain complex behavior [47]. The steps undertaken in this study included the measurement (validity and reliability test) and structural model (hypotheses test, including model fit test) analyses [41, 74].

## Results

### Analysis of the measurement model

Based on construct reliability and validity (convergent and discriminant validity) tests that analyzed the factor loadings, Average Variance Extracted (AVE), Cronbach’s alpha (CA) and Composite Reliability (CR), Dijkstra-Henseler’s rho (RhoA), Fornell-Larcker criterion, and Heterotrait-Monotrait (HTMT) ratio, the items used in this study were good indicators. A full collinearity test was also undertaken to ensure that the model was free from common method bias.

The factor loadings of the items were acceptable with values greater than 0.7 [41–44]. Three items were found to have factor loadings below 0.70 (PFD1: 0.663/ NAT34: 0.647/ NAT38: 0.597) but were retained as the total AVE exceeded 0.50, as Byrne et al.’s (2010) suggestions [23]. Constructs are considered reliable when the CA and CR values are greater than 0.70 [54]. The RhoA for all constructs was above 0.70, indicating that the items were consistently reliable [26]. In addition, AVE values for all constructs also exceeded the 0.50 threshold, affirming strong convergent validity [44]. Table 1 presents the results for the reliability and convergent validity tests.

**Table 1:** Construct Realibilty and Validity

Items	Factor Loadings	CA	rhoA	CR	AVE
**Trust in Key Actors**		0.791	0.792	0.878	0.706
TRUST1: Scientists have done a good job for society.	0.861				
TRUST2: Industries have done a good job for society.	0.838				
TRUST3: Government have done a good job for society.	0.821				
**Attitudes toward Nature versus Materialism**		0.857	0.873	0.881	0.604
NAT1: Society aiming to preserve nature versus society stressing to achieve wealth.	0.644				
NAT2: Society with a centrally planned economy versus society relying on a market-driven economy.	0.846				
NAT3: Society that will stop on development at the expense of any risks versus society deliberately accepting any risks in the attainment of wealth.	0.884				
NAT4: Society that optimizes the protection of the environment above economic growth versus society relying on economic growth above environment protection.	0.894				
NAT5: Society that stress the nature is fragile and easily damaged by human actions versus society which stressing nature can with stand by human actions.	0.556				
**Religiosity**		0.947	0.954	0.956	0.730
REG1: Religion is important in my life.	0.878				
REG2: Religious views are important when I have to make decisions about controversial issues.	0.827				
REG3: Praying is important in my life.	0.912				
REG4: Reading scriptures is important in my life.	0.871				
REG5: Religion is especially important to me because it answers many questions about the meaning of life.	0.893				
REG6: What religion offers me most is comfort when sorrows and misfortune strike.	0.813				
REG7: I try hard to live all my life according to my religious beliefs.	0.799				
REG8: Nothing can occur without God’s involvement in the process.	0.798				
**Perceived Benefits**		0.864	0.868	0.896	0.553
PFD1: technology will enhance the quality of life.	0.657				
PFD2: WiAM technology is useful to the Malaysian society.	0.742				
PFD3: WiAM technology is useful in preventing dengue fever.	0.784				
PFD4: WiAM technology is effective to eradicate dengue.	0.786				
PFD5: WiAM technology is beneficial to me and my family’s health.	0.791				
PFD6: The benefits of the WiAM technology to people outweigh their risks.	0.735				
PFD7: Whatever the risks of the WiAM technology will be dealt with in future research.	0.700				
**Perceived Risks**		0.898	0.903	0.919	0.620
PRD1: Level of worries about the unknown effects of the WiAM technology?	0.756				
PRD2: Any harmful effects from using the WiAM technology will only manifest itself after long term duration?	0.756				
PRD3: WiAM technology pose threat to future generation.	0.820				
PRD4: WiAM technology may give rise to unknown consequences.	0.787				
PRD5: Any danger from the WiAM technology may cause a major catastrophe to Malaysian society.	0.770				
PRD6: How worried are you about the potential risks of the WiAM technology to your health and you family’s health?	0.783				
PRD7: Adverse effects from the WiAM technology are harmful.	0.834				
**Attitudes on WIAM Technique**		0.826	0.828	0.878	0.590
ATW1: WiAM technology should be scaled up.	0.740				
ATW2: Government should provide more financial support to researchers and industries in developing the WiAM technology.	0.754				
ATW3: WiAM technology help government to decrease community’s fatality. (casualities in the community)	0.750				
ATW4: WiAM technology is necessary.	0.800				
ATW5: WiAM technology is encouraged.	0.795				
**Intentions on WIAM Technique**		0.911	0.916	0.931	0.693
INT1: I am willing to support WiAM technology if it can combat dengue.	0.822				
INT2: I am willing to support WiAM technology if it is beneficial to my health and the health of other people.	0.871				
INT3: I am willing to support WiAM technology if there are no other better alternatives.	0.792				
INT4: I am willing to support WiAM technology if there are no other better alternatives.	0.827				
INT5: I am willing to support WiAM technology if they have been proven effective to combat dengue in other areas.	0.868				
INT6: I am willing to support WiAM technology if the government can ensure the effectiveness of it.	0.812				

Discriminant validity is defined as the extent to which the value of the variable significantly differs from other constructs in the model, indicated by the fact that the value of the loading factors in the latent variable is greater [82]. In this study, the Fornell-Larcker criterion and HTMT ratio were used to determine discriminant validity to compare correlations on the square roots of AVE. Each construct has a square root value of AVE higher than other constructs in the Fornell-Larcker criterion test [32]. The HTMT value was the main criterion used to assess discriminant validity [71] and was found to be less than 0.90. Thus, the test meets the criteria [39].

Lohmöller [61] suggested the use of standardized root mean square residual (SRMR) and normed fit index (NFI) as fit measures for PLS-SEM. However, Hair et al. [41] cautioned that the fit criteria for PLS-SEM are still not fully understood as they are at an early stage of development. Nevertheless, both fit indexes were reported for the model as a general guide for the model fit. The SRMR for the model was below 0.08 at 0.073, indicating a good fit [53] (Refer to Table 2). The NFI value for this research model was 0.702, slightly below the 0.9 value recommended by Dijkstra and Henseler [26] and Kim et al. [57]. However, the value is still within an acceptable range and closer to 1. Past studies have reported PLS-SEM models with NFI values above 0.5, a value considered as an acceptable fit [93]. Kock and Lynn [58] proposed that a full collinearity test was undertaken simultaneously to assess lateral and vertical collinearity. The variance inflation factor (VIF) values for all the constructs (factors) were lower than 3.3, suggesting that the study model was free from common method bias (Refer to Table 3).

**Table 2:** Fornell-Larcker Criterion, HTMT, SRMR and NFI

Fornell Larcker Criterion							
	TRUST	NAT	REG	PFD	PRD	ATW	INT
Trust in Key Players	0.840						
Attitudes to Nature vs Materialism	−0.012	0.777					
Religiosity	0.160	−0.152	0.854				
Perceived Benefits	0.383	0.125	0.239	0.744			
Perceived Risks	−0.337	−0.218	− 0.114	−0.349	0.787		
Attitudes on WIAM Technology	0.301	0.023	0.198	0.557	−0.273	0.768	
Intentions on WIAM Technology	0.394	−0.066	0.284	0.519	−0.165	0.676	0.833
**Heterotrait-Monotrait Ratio (HTMT)**							
	TRUST	NAT	REG	PFD	PRD	ATW	INT
Trust in Key Players							
Attitudes to Nature vs Materialism	0.104						
Religiosity	0.199	0.204					
Perceived Benefits	0.458	0.150	0.264				
Perceived Risks	0.393	0.212	0.145	0.390			
Attitudes on WIAM Technology	0.373	0.113	0.224	0.659	0.318		
Intentions on WIAM Technology	0.467	0.108	0.304	0.581	0.179	0.769	
**SRMR Composite Model** = 0.073, **NFI normed fit index** = 0.702

**Table 3:** Results of The Hypothesis Testing

Hypothesis	Path Coefficient	Standard Beta	Standard Error	T-Values	***P***-Values	Decision	VIF	R^**2**^	Q^**2**^	f^**2**^
**H1**	0.285	0.287	0.053	5.377	0.000***	**Supported**	1.153	0.240	0.127	0.093
**H2**	−0.324	−0.327	0.040	8.033	0.000***	**Supported**	1.026	0.172	0.103	0.124
**H3**	0.077	0.078	0.051	1.513	0.065	Not supported				
**H4**	0.111	0.111	0.052	2.124	0.017*	**Supported**	1.092			0.015
**H5**	−0.237	−0.244	0.050	4.773	0.000***	**Supported**	1.024			0.066
**H6**	−0.047	−0.050	0.046	1.023	0.153	Not supported				
**H7**	0.187	0.190	0.047	4.017	0.000***	**Supported**	1.062			0.043
**H8**	−0.099	−0.100	0.047	2.101	0.018*	**Supported**	1.051			0.011
**H9**	0.052	0.049	0.050	1.027	0.152	Not supported				
**H10**	0.493	0.494	0.056	8.788	0.000***	**Supported**	1.316	0.329	0.189	0.275
**H11**	−0.080	−0.080	0.048	1.669	0.048*	**Supported**	1.265			0.007
**H12**	−0.208	−0.206	0.055	3.776	0.000***	**Supported**	1.208			0.047
**H13**	0.676	0.678	0.036	18.705	0.000***	**Supported**	1.000	0.457	0.313	0.841

## Analysis of the structural model

Hair et al. [41] and Ramayah et al. [74] proposed a structural model assessment using the coefficient of determination (R^2^), the predictive relevance (Q^2^), the effect size (f^2^), the beta values (*β*), and *t*-values with an interpretation of the path coefficients on statistical significance (*p*-value). A coefficient of determination (R^2^) value of 0.75 is considered substantial, 0.50 is moderate, while 0.26 is weak [40, 48, 54]. Measuring the degree of the model predictability requires predictive relevance (Q^2^) testing using a blindfolding procedure with an omission distance of 7. The Q^2^ value must be positive and beyond zero [87].

The f^2^ is the effect size of the exogenous construct that impacts the endogenous construct [54]. Cohen [24] denoted that a strong effect size is represented by an f^2^ value of 0.35, 0.15 for medium effect, and 0.02 for a small effect. Hair et al. [44] recommended carrying out bootstrapping with a resample of 5000 as the method to assess R^2^, f^2^, *β-values, t*-values, and *p*-values. The critical *t*-value for a one-tailed test is 1.645 with a significance level of 5% (*p*-values < 0.01).

The R^2^ value for intentions to WiAM technology is 0.457, suggesting that attitude to WiAM technology explains 45.7% of the variance in intentions to adopt WiAM technology (*β* = 0.676, *t* = 18.634, *p* < 0.001, f^2^ = 0.841; large). The Q^2^ value for this model (0.313) was sufficient to support the predictive relevance of the path model for the endogenous construct. These findings supported H13.

Perceived benefits (*β* = 0.493, *t* = 8.788, *p* < 0.001, f^2^ = 0.275) was the most important direct predictor of attitudes to WiAM, followed by perceived risks (*β* = − 0.080, *t* = 1.669, *p* = 0.048, f^2^ = 0.047). Furthermore, 32.9% of the variance in attitudes to WiAM technology was explained by perceived benefit and perceived risk. The Q^2^ value for attitude to WiAM technology was 0.189, which confirmed that the exogenous factors were relevant in predicting the attitude factor. These results supported H10 and H11.

Trust in key players, attitudes to nature versus materialism, and religiosity have a significant positive relationship with perceived benefits, while the perceived risk was negatively associated with perceived benefits. The R^2^ and the Q^2^ values for perceived benefit were 0.240 and 0.127, respectively. The results suggested that 24.0% of the variance in perceived benefit were explained by trust in key players (*β* = 0.285, *t* = 5.377, *p* < 0.001), perceived risk (*β* = − 0.208, *t* = 3.776, *p* < 0.001), attitudes to nature versus materialism (*β* = 0.111, *t* = 2.124, *p* = 0.017), and religiosity (*β* = 0.187, *t* = 4.017, *p* < 0.001). The f^2^ values for the exogenous variables had a small effect on the perceived benefit. Furthermore, the Q^2^ value for perceived benefit indicated that the exogenous variables supported the path on the predictive model. Hence, the findings supported H1, H4, and H7.

Perceived risk (R^2^ = 0.172) was weakly explained by trust in key players (*β* = − 0.324, *t* = 8.033, *p* < 0.001), attitudes to nature versus materialism (*β* = − 0.237, *t* = 4.773, *p* < 0.001), and religiosity (*β* = − 0.099, *t* = 2.101, *p* = 0.018). These factors were negatively influenced and explained 17.2% of the variance in perceived risks. The f^2^ values for the exogenous variables had a small effect on the perceived risk. The Q^2^ value for perceived risk was 0.103, indicating that the exogenous factors were relevant to predicting perceived risk in this research model. These results supported H2, H3, H8, and H12.

## Discussion and implications

Attitudes had a strong positive association with intention to support the use of WiAM in this study. Krishnan and Rahim [59] emphasized that perceptions and attitudes toward a health issue will influence an individual’s intentions to act on this issue. Additionally, perceived benefits of WiAM were the most important direct predictor of attitudes to WiAM, followed by perceived risks. The results further explain the findings of Arham et al. [12, 13], who reported that Malaysian stakeholders perceived high benefits and were highly positive toward WiAM technology while concurrently acknowledging the risk.

Perceived benefits and risks were also found to be inversely related. Mustapa et al. [67] identified significant relationships between perceived benefits and risks on intentions to adopt new forms of medical technology, such as nutrigenomics, and inverse relationships between both factors. However, other studies reported a positive influence of perceived benefits on attitudes toward genetically modified mosquitoes, but not perceived risks [8]. Both factors were unrelated, although other studies have identified an inverse relationship between the two factors in medical technology [66, 67].

Trust in key players, attitudes toward nature versus materialism, and religiosity positively influenced the perceived benefits and negatively influenced the perceived risks. The findings indicated that stakeholders trust those playing a crucial role in dengue control, are highly committed to their religion, and are more inclined toward materialism. They will perceive higher benefits and fewer risks, which translates into a positive attitude toward WiAM technology. Amin and Hashim [8] also reported that trust in crucial players was positively associated with perceived benefits and attitudes toward genetically modified mosquitoes. Additionally, the findings aligned with Trumbo and McComas [88], who discovered that the key players’ role influences a person’s technology acceptance. Thus, when stakeholders have high trust in key players, they tend to focus on WiAM benefits. According to Bronfman et al. [22], stakeholders can accept the risks arising from new technologies when they believe that certain parties are responsible for managing the risks.

Attitudes toward nature versus materialism or societal values reflected the respondents’ tendency to conserve nature or focus on artificial materials [37]. In this study, stakeholders inclined toward materialism tend to perceive more benefits and fewer risks from WiAM technology. These findings are supported by Amin and Hashim [8], who found that stakeholders who tend to be materialistic perceive the benefits of genetically modified mosquitoes more than the perceived risks.

Importantly, this study also showed that when stakeholders are committed to their religion, they tend to rate the WiAM technology as less risky and beneficial in controlling dengue. This finding is reinforced by studies conducted by Amin et al. [5, 6], Amin and Hashim [8], Arham et al. [12, 13], and Mustapa et al. [67]. The researchers found that Malaysian stakeholders who were deeply attached to their religion were positive toward new technologies such as WiAM, probably because the technology was perceived as acceptable in their religion. Furthermore, most study respondents were Malay and Muslims. The Islamic Law (Maqasid Syariah) outlines the need to preserve health and life as one of its five objectives [80]. Islam encourages science and technology, provided such applications bring benefits (*maslahah*) and minimizes harm (*mafsadah*) to society and the environment [80]. This concept explains the positive relationships between religiosity and perceived benefits.

**Fig. 2:**
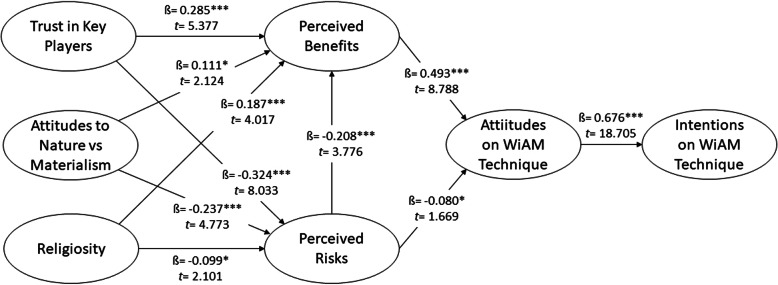
Model for Stakeholders’ Attitudes and Intentions toward Supporting the Use of WiAM Technology

Therefore, the study model helps identify the predictors that can serve as valuable indicators for scientists, governments, and policymakers regarding the mass introduction of WiAM in Malaysia and other countries with a similar culture. However, recognizing several limitations of the model related to sampling, measurement scales, and time is crucial. The final model presented in Fig. 2 is valid for illustrating the stakeholders’ attitudes and intentions toward supporting WiAM usage.

However, the model cannot necessarily be generalized to the entire Malaysian population because the data was only collected from the Klang Valley region. The respondents comprised only two stakeholders’ groups: scientists and the public. Therefore, the sampling area in future research should be extended to other Malaysian regions and expanded to include other stakeholders, such as policymakers, media, religious experts, and non-governmental organizations (NGOs). The comprehensive research would fully reflect the range of attitudes across the country.

Furthermore, the model was developed using selected variables based on previous findings that may not be exhaustive. Thus, the influence of other variables should also be considered. Additionally, the effects of the identified variables may vary over time. Therefore, longitudinal research should be performed to assess these effects.

## Conclusion

The study findings are vital in determining the factors influencing attitudes and intentions regarding community and expert acceptance of WiAM technology. This study is the first to develop the SEM model of attitudes and intentions to adopt WiAM technology that has successfully identified the significant predictors. The findings affirmed the multi-faceted nature of stakeholders’ attitudes and intentions to support the WiAM usage. These findings may serve as a meaningful benchmark for social acceptance when making decisions related to utilizing WiAM technology as a method to control dengue. The WIAM technology has enormous potential to enhance public health quality and the environment.

## Supplementary Information

**Additional file 1.** A survey on dengue prevention technique and control in Klang Valley, Malaysia.

## Data Availability

All relevant data are within the manuscript, the datasets are also available in the Mendeley Data repository, https://data.mendeley.com/datasets/4ky5krhf37/1 and the English version questionnaire was included in supplementary file.

## Abbreviations

**APVMA**: Australian pesticides and veterinary medicines authority
**ATW**: Attitudes on WIAM technique
**AVE**: Average variance extracted
**CA**: Cronbach’s alpha
**CR**: Composite reliability
**f**^**2**^: Effect size
**HTMT**: Heterotrait-Monotrait ratio
**INT**: Intentions on WIAM technique
**NAT**: Attitudes to Nature vs Materialism
**NFI**: Normed fit index
**NGO**: Non-governmental organization
**PFD**: Perceived benefits
**PLS-SEM**: Partial least square-structural equation modeling
**PRD**: Perceived risks
**Q**^**2**^: Predictive relevance
**R**^**2**^: Coefficient of determination
**REG**: Religiosity
**rhoA**: Dijkstra-Henseler’s rho
**SEM**: Structural equation modeling
**SRMR**: Standardized root mean residual
**TRUST**: Trust in key players
**VIF**: Variance inflation factor
**WiAM**: *Wolbachia*-infected Aedes mosquito
