# Supplementary material for: Determinants of stakeholders’ attitudes and intentions toward supporting the use of Wolbachia-infected Aedes mosquitoes for dengue control
Source: BMC Public Health. 2021 Dec 23;21:2314. doi: 10.1186/s12889-021-12166-w (PMC8697497; doi:10.1186/s12889-021-12166-w)
Supplement: Supplementary file 1 — Additional file 1. A survey on dengue prevention technique and control in Klang Valley, Malaysia. [file 12889_2021_12166_MOESM1_ESM.docx]

***
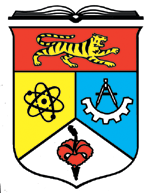
*UNIVERSITI KEBANGSAAN MALAYSIA**

**43600 UKM, BANGI**

**SELANGOR DARUL EHSAN**

**A SURVEY ON DENGUE PREVENTION TECHNIQUE AND CONTROL IN KLANG VALLEY, MALAYSIA**

National University of Malaysia is conducting a survey regarding the level of awareness and public perception towards the *Wolbachia*-infected Aedes Mosquitoe (WiAM) technology to the environment. We appreciate your cooperation in helping us to complete the survey form as attached. All information used is for research purposes and are kept confidential.

Thank you very much for your cooperation.

Prof. Dr. Latifah Amin (Project Leader)

**A SURVEY ON DENGUE PREVENTION TECHNIQUE AND CONTROL IN MALAYSIA**

| **INSTRUCTION:** Please circle the number that corresponds to your answer for each question. Your private information will be strictly preserved.  **Example:** Do you find modern biotechnology useful to society?   \| Strongly Strongly  disagree agree \| \| --- \| \| **1 2 3 4 5 6 7** \| |
| --- | --- | --- |

**SECTION A**

Please circle the number that corresponds to your answer for each question.

**PERCEIVED BENEFITS**

| Wolbachia bacteria is able to shorten the lifespan of Aedes mosquitoes. | Strongly Strongly  disagree agree |
| --- | --- |
| Q1. The following method will enhance the quality of life. | **1 2 3 4 5 6 7** |
| Q2. The following method is useful to the Malaysian society. | **1 2 3 4 5 6 7** |
| Q3. The following method is useful in preventing dengue fever. | **1 2 3 4 5 6 7** |
| Q4. The following method is effective to eradicate dengue. | **1 2 3 4 5 6 7** |
| Q5. The following method is beneficial to me and my family’s health. | **1 2 3 4 5 6 7** |
| Q6. The benefits of the following method to people outweight their risks. | **1 2 3 4 5 6 7** |
| Q7. Whatever the risks of the following method will be deal with future research. | **1 2 3 4 5 6 7** |

**PERCEIVED RISKS**

| Wolbachia bacteria is able to shorten the lifespan of Aedes mosquitoes. | Strongly Strongly  disagree agree |
| --- | --- |
| Q1. Level of worries about the unknown effects of the following method? | **1 2 3 4 5 6 7** |
| Q2. Any harmful effects from using the following method will only manifest itself after long term duration? | **1 2 3 4 5 6 7** |
| Q3. Using the following method, they pose threat to future generation. | **1 2 3 4 5 6 7** |
| Q4. The following method may give rise to unknown consequences. | **1 2 3 4 5 6 7** |
| Q5. Any danger from the following method may cause a major catastrophe to Malaysian society. | **1 2 3 4 5 6 7** |
| Q6. How worried are you about the potential risks of the following method to your health and you family’s health? | **1 2 3 4 5 6 7** |
| Q7. Adverse effects from the following method is harmful. | **1 2 3 4 5 6 7** |

**ATTITUDES ON WIAM TECHNIQUE**

| Wolbachia bacteria is able to shorten the lifespan of Aedes mosquitoes. | Strongly Strongly  disagree agree |
| --- | --- |
| Q1. The following method should be scaled up. | **1 2 3 4 5 6 7** |
| Q2. Government should provide more financial support to researchers and industries in developing the following method. | **1 2 3 4 5 6 7** |
| Q3. The following method helps government to decrease community’s fatality. (casualities in the community) | **1 2 3 4 5 6 7** |
| Q4. The following method is necessary. | **1 2 3 4 5 6 7** |
| Q5. The following methods is encouraged. | **1 2 3 4 5 6 7** |

**TRUST IN KEY PLAYERS**

| The following institutions/groups have done a good job for the society? | Not Very  good good  at all |
| --- | --- |
| Q1. Scientists / Researchers from Universities and Research Institution. | **1 2 3 4 5 6 7** |
| Q2. Pesticides and Pharmaceutical Industries. | **1 2 3 4 5 6 7** |
| Q3. Government sector on regulations such as Ministry of Health and Biosafety Department. | **1 2 3 4 5 6 7** |

**ATTITUDES TO NATURE VERSUS MATERIALISM**

| Perspective A | Preferred Preferred  to A to B  **1 2 3 4 5 6 7** | Perspective B |
| --- | --- | --- |
| 1a) A society aiming at preserving nature to its original state. | **1 2 3 4 5 6 7** | 1b) A society stressing the use of the nature in order to achieve wealth. |
| 2a) A society with a centrally planned economy. | **1 2 3 4 5 6 7** | 2b) A society relying on a market-driven economy. |
| 3a) A society that will stop on development at the expense of any risks. | **1 2 3 4 5 6 7** | 3b) A society deliberately accepting any risks in the attainment of wealth. |
| 4a) A society that optimizes the protection of the environment above economic growth. | **1 2 3 4 5 6 7** | 4b) A society that optimizes economic growth above environment protection. |
| 5a) A society that stressing that nature is fragile and easily damaged by human actions. | **1 2 3 4 5 6 7** | 5b) A society that stressing nature can with stand human actions. |

**ATTITUDES TOTECHNOLOGY**

|  | Strongly Strongly  disagree agree |
| --- | --- |
| Q1. Modern technology has made human lost respect towards nature | **1 2 3 4 5 6 7** |
| Q2. The development of science and technology has made human more focused on profits rather than the development of nature. | **1 2 3 4 5 6 7** |
| Q3. The over-reliance of human on modern technology has destroyed humanistic values. | **1 2 3 4 5 6 7** |
| Q4. Relentless science and technological development will ultimately lead to humaneness extermination. | **1 2 3 4 5 6 7** |
| Q5. Modern technology has upset the balance of nature | **1 2 3 4 5 6 7** |
| Q6. The existence of industry and technology to urban life has contributed to more problems and worries. | **1 2 3 4 5 6 7** |

**INTENTIONS ON WIAM TECHNIQUE**

|  | Strongly Strongly  disagree agree |
| --- | --- |
| Q1. I am willing to support WiAM technology if it can combat dengue. | **1 2 3 4 5 6 7** |
| Q2. I am willing to support WiAM technology if it is beneficial to my health and the health of other people. | **1 2 3 4 5 6 7** |
| Q3. I am willing to support WiAM technology if there are no other better alternatives. | **1 2 3 4 5 6 7** |
| Q4. I am willing to support WiAM technology if there are no other better alternatives. | **1 2 3 4 5 6 7** |
| Q5. I am willing to support WiAM technology if they have been proven effective to combat dengue in other areas. | **1 2 3 4 5 6 7** |
| Q6. I am willing to support WiAM technology if the government can ensure the effectiveness of it. | **1 2 3 4 5 6 7** |

**RELIGIOSITY**

|  | Strongly Strongly  disagree agree |
| --- | --- |
| Q1. Religion is important in my life. | **1 2 3 4 5 6 7** |
| Q2. Religious views are important when I have to make decisions about controversial issues. | **1 2 3 4 5 6 7** |
| Q3. Praying is important in my life. | **1 2 3 4 5 6 7** |
| Q4. Reading scriptures is important in my life. | **1 2 3 4 5 6 7** |
| Q5. Religion is especially important to me because it answers many questions about the meaning of life. | **1 2 3 4 5 6 7** |
| Q6. What religion offers me most is comfort when sorrows and misfortune strike. | **1 2 3 4 5 6 7** |
| Q7. I try hard to live all my life according to my religious beliefs. | **1 2 3 4 5 6 7** |
| Q8. Nothing can occur without God’s involvement in the process. | **1 2 3 4 5 6 7** |

**SECTION B:**

The answers to following questions will be used only for general analysis. Your personal information will be confidential.

Q1. **Gender:** 1. Male 2. Female

Q2. **Types of Stakeholders**: 1. Scientists 2. Public

Q3. **Sector of Occupations**: 1. Government 2. Non-Government

Q4. **Religion**: 1. Islam 2. Other religions

Q5. **Nation**: 1. Malay 2. Chinese 3. Indian 4. Others

Q6. **Age**: 1. 18-28 years old 2. 29-39 years old 3. 40 years and above

Q7. **Level of education**: 1. High School and Pre-University 2. Diploma 3. Degree 4. Master and PhD

**THANK YOU**
